# A survey around the Italian pediatric units on current clinical practice for Sleep Disordered Breathing (SDB)

**DOI:** 10.1186/s13052-019-0658-2

**Published:** 2019-06-26

**Authors:** L. Nosetti, M. G. Paglietti, L. Brunetti, L. Masini, S. La Grutta, G. Cilluffo, M. Zaffanello, E. Verrillo, M. Pavone, A. C. Niespolo, G. Broggi, R. Cutrera, the Italian Pediatric Respiratory Diseases Society (SIMRI) Sleep Disordered Breathing Working Group

**Affiliations:** 1 Pediatric Clinic University of Insubria, Via Ravasi, 2, 21100 Varese, Italy; 2 Sleep and Long Term Ventilation Unit, Pediatric Pulmonology & Respiratory Intermediate Care Unit, Academic Department of Pediatrics (DPUO), Bambino Gesù Children’s Hospital, IRCCS, Rome, Italy; 3 U.O.C. Pediatria Dip. Materno-Infantile, Az. Osp. Ente Ecc. Pia Fondazione di Cura e Religione “Card. G. Panico” Tricase, via S. Pio X, 4, 37100 Tricase (Lecce), Italy; 4 Department of Pediatrics, Santobono Children’s Hospital, AORN Santobono-Pausilipon, Pediatric Pulmonology & Respiratory Intermediate Care Unit, , Naples, Italy; 5 National Research Council (CNR) Institute of Biomedicine and Molecular Immunology “Alberto Monroy” IBIM, Via Ugo La Malfa, 153, 90146 Palermo, Italy; 6 Pediatric Clinic, University of Verona, Hospital for Women and Children, Piazzale Aristide Stefani, 1, 3716 Verona, Italy; 7 Bocconi University, Via Roberto Sarfatti, 25, 20136 Milan, Italy

**Keywords:** Sleep disordered breathing, Children, Survey, Italy, Pediatric units

## Abstract

**Background:**

During recent years, interest on Sleep Disordered Breathing (SDB) in pediatric age has increased, due to the impact on quality of life, psycho-physical attitude and other serious morbidities if undiagnosed and untreated.

**Methods:**

Italian Pediatric Respiratory Diseases Society (SIMRI) SDB-Working Group carried out an exploratory survey in Italy, from January to December 2016, to assess the diagnostic and therapeutic pathways, perception and relevance of SDB in Italian Hospitals.

**Results:**

A questionnaire was sent to 180 Pediatric Units (PUs) distributed throughout the Italy; 102 Pediatric Units (PUs; 56.6%) answered and among them 57% dealt with SDB, and 94% recognized SDB as a major problem. Instrumental tests performed by the PUs were saturimetry (66%), nocturnal polygraphy with complete cardio-respiratory monitoring (46%) and full polysomnography (23%). In addition, hospital pediatricians reported that 54% of parents were unaware of the SDB and 84% did not know their complications. In the Northern Italy, the diagnosis was frequently performed with instrumental tools and the treatment was often surgical. In the Southern Italy the diagnosis was clinical, and the treatment was usually with drugs.

**Conclusions:**

The results of our study showed a heterogeneity in the diagnosis and treatment of SDB throughout Italy. Parents know little about SDB and their complications. The operator satisfaction was associated with the availability of tools for diagnosing SDB.

**Electronic supplementary material:**

The online version of this article (10.1186/s13052-019-0658-2) contains supplementary material, which is available to authorized users.

## Introduction

“Sleep Disordered Breathing” (SDB) is a spectrum of disorders characterized by snoring and/or increased respiratory effort due to increased airway resistance and pharyngeal collapse and include [1]:

- Primary Snoring
- Obstructive hypoventilation
- Upper Airway Resistance Syndrome (UARS)
- Obstructive Sleep Apnea Syndrome (OSAS)

SDB is frequent in children, although it is often underestimated, and its impact on the overall childhood health is far from irrelevant: it ranks, in fact, at the third place in the classification of the factors that threaten the health during developmental age [2].

The experience of the clinicians is one of the fundamental pillars of evidence-based practice to improve the quality of care. No studies on the management of pediatric SDB in Italy were performed so far.

Therefore, this cross-sectional exploratory survey is aimed to evaluate, in the Italian Pediatric Units (PUs), the knowledge of the problem, the diagnostic approaches, the therapeutic interventions and the overall satisfaction of pediatricians about SDB management.

## Materials and methods

The Italian Pediatric Respiratory Diseases Society (SIMRI) SDB Working Group carried out an e-mail-based exploratory survey, aimed at assessing the awareness, attitude, practice and satisfaction on SDB in children. The study is designed in 3 phases.

### First phase

SDB Working Group developed a simple questionnaire not yet validated. The questionnaire was costructed by adapting other existing and validated questionnaires for assessing sleep knowledge in a medical education setting [3–5].

This questionnaire has been drawn up, comprising 11 easy-to-fill questions for the Italian PUs. The questionnaire (attached Additional file 1), excluding demographic data, was structured in four main sections: Awareness about SDB burden (section A), Attitude concerning the workout for SDB diagnosis (section B), Practice on the SDB treatment approach (section C), Satisfaction assessing feelings of personal competence about the management of SDB (section D).

### Second phase: data collection

The data collection phase was performed from January to December 2016.

The questionnaire was sent by e-mail to 180 PUs in Italy and re-mailed after 3 months to the PUs who had not previously answered. The 180 PUs were researched through the Federazione Italiana delle Associazioni e Società Scientifiche dell’Area Pediatrica (FIARPED, http://www.fiarped.it/go/mission).

### Third phase: data processing

Data were presented as n (%). Differences of categorical variables were analyzed using Chi-squared test. Analyses were performed using R 3.4.2 software. A *p*-value< 0.05 was considered statistically significant.

## Results

### Awareness, attitude and satisfaction

**Fig. 1:**
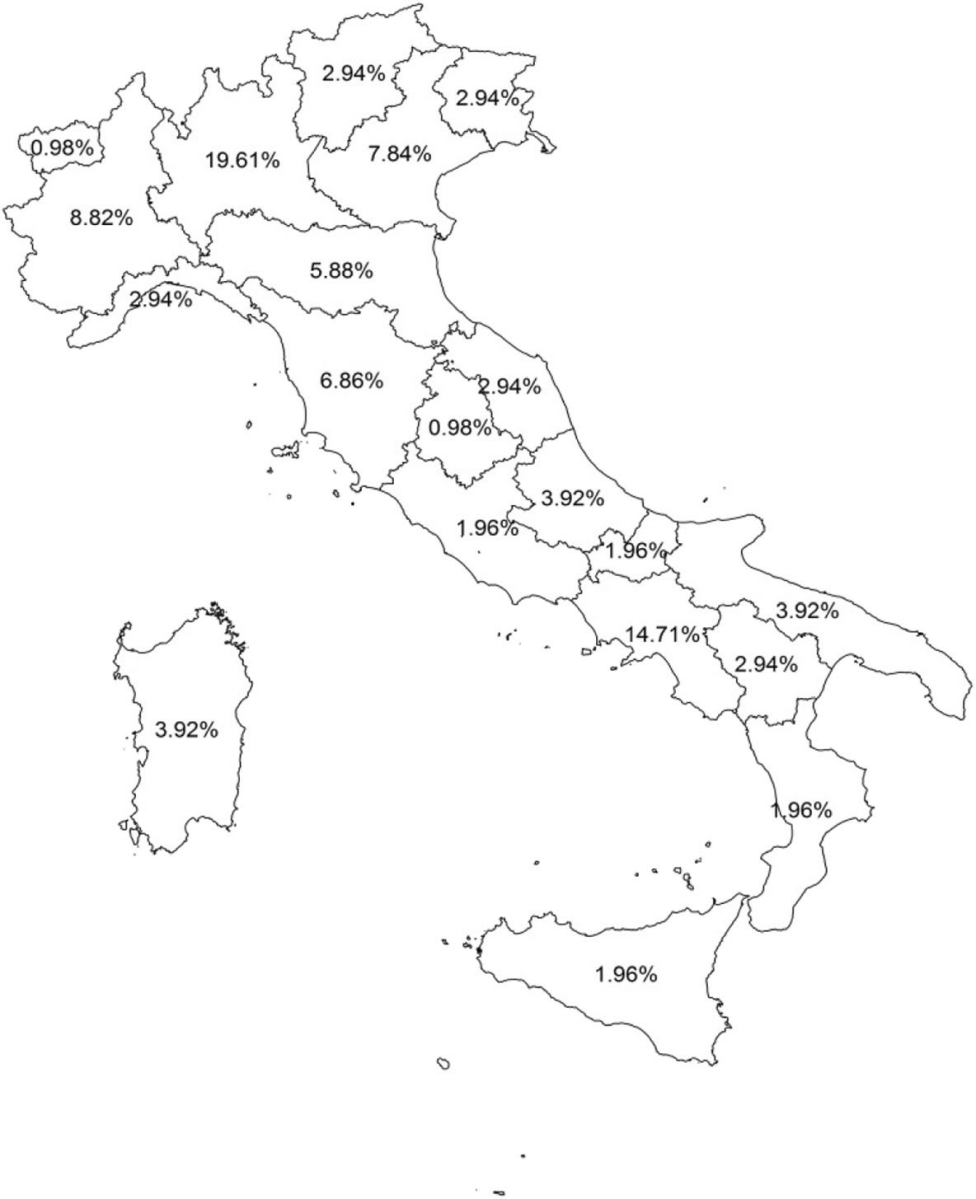
Geographical distribution of the PUs respondents

A total of 102 Italian PUs completed the questionnaire (out of 180, 56.6%); A total of 53 PUs in the Northern Italy (51.9%), 13 in Center Italy (12.7%) and 36 in the Southern Italy (35.2%) reply to the questionnaire (Fig. 1). Just over half of those who answered considered SDB in clinical practice. Most of those who answered thought that SDB was relevant or very relevant problem. Just under half of PUs reported that parents were informed on SDB condition but only a minority reported that parents knew of potential serious complications of SDB. Most respondents made SDB diagnosis using both clinical and instrumental tools. Just under half managed SDB used pharmacological treatment, half suggested adenoidectomy, much less than half suggested adenotonsillectomy, half recommended weight loss and just under half never suggested non-invasive ventilation (NIV). Regarding instrumental tools, much more than half of the PUs performed night pulse oximetry, just under half used polygraphy with EEG and just under a quarter used full polysomnography (PSG) (Table 1). Regarding satisfaction, half of those who answered were satisfied about SDB management. Operator satisfaction correlated with the availability of instrumental diagnosis.

**Table 1:** Awareness, attitude and satisfaction of SDB management

	*n*=102
Section A	
Do you consider SDB in your clinical practice?	58 (57%)
Do you think SDB is a problem:	
Very relevant	44 (43%)
Relevant	52 (51%)
Little relevant	6 (6%)
Are parents informed about SDB?	47 (46%)
The family of children with SDB are aware of potential serious complications of SDB?	16 (16%)
Section B	
How do you make SDB diagnosis	
clinical evidence	32 (31%)
clinical evidence and instrumental measurements	70 (69%)
Section C	
Managing patient with SDB, how often do you propose the following treatment	
Drugs:	
never	17 (17%)
rarely	32 (31%)
often	46 (45%)
very often	7 (7%)
Adenoidectomy	
never	8 (8%)
rarely	42 (41%)
often	50 (49%)
very often	2 (2%)
Adenotonsillectomy	
never	6 (6%)
rarely	51 (50%)
often	41 (40%)
very often	4 (4%)
Weight loss	
never	5 (5%)
rarely	37 (36%)
often	52 (51%)
very often	8 (8%)
Non-invasive ventilation	
never	49 (48%)
rarely	43 (42%)
often	8 (8%)
very often	2 (2%)
Section D	
Are you satisfied how you managed SDB patients?	51 (50%)
Do you perform the night pulse oximetry tests on a child	67 (66%)
Do you perform the poligraphy with monitoring complete cardiorespiratory tests on a child	47 (46%)
Do you perform the complete polysomnography with EEG tests on a child	23 (23%)

### Geographical differences

Differences among Northern, Central and Southern Italy are reported in Table 2. Pediatricians of Northern Italy much more than half thought that much less than half parents were informed about SDB than pediatrician living in Central and Southern Italy (just over half). A clinical and instrumental diagnosis was performed much more than half by Pediatricians of Northern and in all Central Italy than pediatricians living in Southern of Italy (just over half). Other differences between the geographical areas regarded treatments. In particular, drugs were frequently proposed in both Central and Southern (just over half) than in Northern Italy (much less than half). Adenotonsillectomy is more frequently proposed by pediatricians living at Central Italy (much more than half) and North Italy (just under half). No differences in satisfaction were observed between geographical areas.

**Table 2:** Awareness, attitude and satisfaction of SDB management given geographical area

	North *n*=53	Center *n*=13	South *n*=36	*p*-value
Section A				
Do you consider SDB in your clinical practice?	31 (58%)	10 (77%)	17 (47%)	0.191
Do you think SDB is a problem:				0.621
Very relevant	21 (40%)	7 (54%)	16 (44%)	
Relevant	30 (57%)	5 (38%)	17 (47%)	
Little relevant	2 (4%)	1 (8%)	3 (8%)	
Are parents informed about SDB?	32 (60%)	4 (31%)	11 (31%)	**0.011**
The family of children with SDB are aware of potential serious complications of SDB?	10 (19%)	1 (8%)	5 (14%)	0.697
Section B				
How do you make SDB diagnosis				**0.011**
clinical evidence	17 (32%)	0 (0%)	15 (42%)	
clinical evidence and instrumental measurements	36 (68%)	13 (100%)	21 (58%)	
Section C				
Managing patient with SDB, how often do you propose the following treatment				
Drugs :				**0.001**
never	6 (11%)	0 (0%)	11 (31%)	
rarely	24 (45%)	3 (23%)	5 (14%)	
often	20 (38%)	7 (54%)	19 (53%)	
very often	3 (6%)	3 (23%)	1 (3%)	
Adenoidectomy				0.191
never	4 (8%)	3 (23%)	1 (3%)	
rarely	21 (40%)	4 (31%)	17 (47%)	
often	27 (51%)	5 (38%)	18 (50%)	
very often	1 (2%)	1 (8%)	0 (0%)	
Adenotonsillectomy				**0.032**
never	4 (8%)	0 (0%)	2 (6%)	
rarely	24 (45%)	3 (23%)	24 (67%)	
often	23 (43%)	8 (62%)	10 (28%)	
very often	2 (4%)	2 (15%)	0 (0%)	
Weight loss				0.461
never	2 (4%)	0 (0%)	3 (8%)	
rarely	18 (34%)	7 (54%)	12 (33%)	
often	30 (57%)	4 (31%)	18 (50%)	
very often	3 (6%)	2 (15%)	3 (8%)	
Non-invasive ventilation				0.363
never	27 (51%)	5 (38%)	17 (47%)	
rarely	22 (42%)	5 (38%)	16 (44%)	
often	2 (4%)	3 (23%)	3 (8%)	
very often	2 (4%)	0 (0%)	0 (0%)	
Section D				
Are you satisfied how you managed SDB patients?	29 (55%)	8 (62%)	14 (39%)	0.217
Do you perform the night pulse oximetry tests on a child	35 (66%)	11 (85%)	21 (58%)	0.219
Do you perform the poligraphy with monitoring complete cardiorespiratory tests on a child	26 (49%)	7 (54%)	14 (39%)	0.571
Do you perform the complete polysomnography with EEG tests on a child	15 (28%)	2 (15%)	6 (17%)	0.449

### Satisfied vs not satisfied pediatricians

Differences between satisfied and not satisfied pediatricians are reported in Table 3. Satisfied pediatricians considered SDB much more than half in their clinical practice than not satisfied. Satisfied pediatricians thought that a quarter of parents of children with SDB better know the serious complications from untreated SDB than not satisfied. Satisfied were able to diagnose SDB using both clinical and instrumental diagnosis (much more than half) than not-satisfied (half). No differences in management of children with SDB were found. Satisfied pediatricians performed night pulse oximetry (much more than half), poligraphy with EEG (much more than half) and full polysomnography (much less than half) than not satisfied.

**Table 3:** Awareness and attitude by satisfaction

	Not satisfied	Satisfied	*p*-value
	*n*=51	*n*=51	
Section A			
Do you consider SDB in your clinical practice?	20 (39%)	38 (75%)	**<0.001**
Do you think SDB is a problem:			0.561
Very relevant	20 (39%)	24 (47%)	
Relevant	4 (8%)	2 (4%)	
Little relevant	27 (53%)	25 (49%)	
Are parents informed about SDB?	21 (41%)	26 (51%)	0.427
The family of children with SDB are aware of potential serious complications of SDB?	3 (6%)	13 (25%)	**0.012**
Section B			
How do you make SDB diagnosis			**0.0002**
clinical evidence	25 (49%)	7 (14%)	
clinical evidence and instrumental measurements	26 (51%)	44 (86%)	
Section C			
Managing patient with SDB, how often do you propose the following treatment			
Drugs :			0.086
never	13 (25%)	4 (8%)	
rarely	15 (29%)	17 (33%)	
often	21 (41%)	25 (49%)	
very often	2 (4%)	5 (10%)	
Adenoidectomy			0.354
never	3 (6%)	5 (10%)	
rarely	24 (47%)	18 (35%)	
often	24 (47%)	26 (51%)	
very often	0 (0%)	2 (4%)	
Adenotonsillectomy			0.639
never	3 (6%)	3 (6%)	
rarely	24 (47%)	27 (53%)	
often	23 (45%)	18 (35%)	
very often	1 (2%)	3 (6%)	
Weight loss			0.249
never	1 (2%)	4 (8%)	
rarely	6 (12%)	2 (4%)	
often	20 (39%)	17 (33%)	
very often	24 (47%)	28 (55%)	
Non-invasive ventilation			0.050
never	31 (61%)	18 (35%)	
rarely	1 (2%)	1 (2%)	
often	16 (31%)	27 (53%)	
very often	3 (6%)	5 (10%)	
Section D			
Do you perform the night pulse oximetry tests on a child	26 (51%)	41 (80%)	**0.003**
Do you perform the poligraphy with monitoring complete cardiorespiratory tests on a child	12 (24%)	35 (69%)	**<0.001**
Do you perform the complete polysomnography with EEG tests on a child	6 (12%)	17 (33%)	**0.016**

## Discussion

The first important result of our study was to obtain medical information on SDB management and satisfaction among Italian macro-regions: 52% of the data come from the Northern, 13% from the Center, and 35% from the Southern of Italy. Overall, our results were similar to other online survey performed in USA to evaluate the current practice patterns of pediatric otolaryngologist [6].

Fifty-seven percent of the interviewed PUs dealt with SDB; 51% recognized that SDB was a significant problem in pediatric age and 43% a very important problem. However, awareness of the problem was not similar among parents. According to the interviewed PUs, 46% of parents recognized SDB, but only 16% were informed about their serious complications. The most awarded parents lived in the Northern.

Regarding how the diagnosis is performed, 69% of the interviewed Centers had instrumental tests. In particular, in order of decreasing frequency, the overnight oximetry was used in 66% of the Centers, nocturnal polygraphy in 46% of the Centers and the full PSG in 23%. It is well known that full PSG is the gold standard in SDB diagnosis [7] but was available from few Center being expensive and time-consuming. In agreement with OSAS guidelines, when full PSG is not available, it is advisable to use easier instrumental monitoring techniques [8, 9].

Italian centers that dealt with SDB, which also had the possibility to perform instrumental diagnosis (73%), had a greater perception of the seriousness of the problem: in 99% of cases they considered SDB a serious or very serious problem, against 84% of Centers that do not dealt with SDB. The therapeutic approach most often proposed for SDB management was weight loss, followed by pharmacological treatment, adenoidectomy, adenotonsillectomy and NIV. These data only partially confirmed the literature [10] showing that morbid obese OSAS phenotype is increasing by time, particularly in school-aged and teen-aged children [11] being the strongest risk factor for mild and moderate SDB [12]. Obesity and OSAS appeared to contribute each other on initiation and progression, promoting the onset and worsening of metabolic dysfunction. Both conditions can be viewed as low-grade inflammatory disorders. Weight loss is a hard-to-obtain strategy and whose efficacy has yet to be demonstrated in pediatric age. International literature shows that adenotonsillectomy is the main therapeutic choice, although adenotonsillectomy is less effective in obese patients [13].

Regarding the level of satisfaction of the interviewed pediatricians, 50% were satisfied about SDB management.

Regarding parameters that can increase the chances of being satisfied, we recognized the availability of instrumental diagnosis.

Centers with instrumental diagnostic approaches propose multidisciplinary courses, visit more children over time and carry out more diagnoses of OSAS. While there are differences in the various diagnostic management, absence of difference about hospital operators’ satisfaction in Italian macro-regions could be due to the reduced perception of the problem and the scarce possibility of diagnosis.

In USA SDB education campaign can improve health care outcomes and reduce medical costs [14]. The possibility of a diagnostic work-up may significantly reduce the costs directly associated with untreated SDB. Recognition and treatment of SDB is crucial for health and wellbeing of children, mainly when SDB is a comorbidity [15–17].

Main limitation of the present investigation was the achievement of only 56% of response rate. We are convinced that the sample size is limited to the responses received, despite to many solicitations. Consequently, the failure of the reply by PUs investigated may suggested the unavailability of a diagnostic work-up for SDB investigation and management. We do not have clear information about the type of PUs offering sanitary specialties among those that have not responded.

Therefore, this is the first survey on the recognition and treatment of childhood SDB in Italy.

## Conclusions

The study was aimed to assess the overall knowledge and the degree of satisfaction of PUs about the diagnostic and therapeutic management of SDB in Italy. It is an interesting picture of the Italian condition. Heterogeneous reality emerged on the diagnostic-therapeutic domain in the perception of SDB in childhood. Not all Italian PUs know the problem of pediatric SDB. Therefore, to improve their preparation, participation in distance or meeting training could be useful. This is the objective of the SIMRI SDB Working Group through the publication of informative material and the training of health personnel competent for the management of pediatric SDBs.

The project will continue with the administration of a similar questionnaire to the General Pediatricians throughout the national territory, to test the knowledge of pediatric SDB. It is necessary to expand the knowledge of SDB in the Italian PUs.

Diagnosis and treatment pathways for SDB is recommended, making local and territory-based network and focusing on high quality and specialized PUs. The economic benefit related to the high number of patients treated from few specialized PUs increase the efficiency and creates learning-by-practice of medical staff.

## Additional file

Additional file 1: SleepPed questionnaire. (DOCX 48 kb)

## Abbreviations

NIV: noninvasive ventilation
OSAS: Obstructive Sleep Apnea Syndrome
PSG: polysomnography
PUs: Pediatric Units
SDB: sleep disordered breathing
UARS: Upper Airway Resistance Syndrome
