# Supplementary material for: A survey around the Italian pediatric units on current clinical practice for Sleep Disordered Breathing (SDB)
Source: Ital J Pediatr. 2019 Jun 26;45:75. doi: 10.1186/s13052-019-0658-2 (PMC6595596; doi:10.1186/s13052-019-0658-2)
Supplement: Supplementary file 1 — SleepPed questionnaire. (DOCX 48 kb) [file 13052_2019_658_MOESM1_ESM.docx]

***SleepPed questionnaire***

*created by Sleep Disorder Working Group of (SDWG) Italian Pediatric Respiratory Society (SIMRI)*

**Demographic data**

*1) From which region do you come from:*

Abruzzo Basilicata Calabria Campania Emilia-Romagna Friuli-Venezia Giulia Lazio Liguria

Lombardia Marche Molise Piemonte Puglia Sardegna Sicilia Trentino-Alto Adige Umbria

Valle d'Aosta Veneto

**Section A**

2) *Sleep disordered breathing are currently considered in your current clinical practice?* Yes No

3) *Do you think SDB is a problem:*

- not relevant
- little relevant
- relevant
- very relevant

4) *In your opinion, are parents informed about the existence of SDB?* Yes No

5) *According to you, the family of children with SDB are aware of potential serious complications of SDB?*
 Yes No

**Section B**

6) *How do you make SDB diagnosis*

- clinical evidence
- clinical evidence and instrumental measurements

**Section C**

7) *Managing patient with SDB, how often do you propose the following treatment*

Drugs :

- never
- rarely
- often
- very often

Adenoidectomy

- never
- rarely
- often
- very often

Adenotonsillectomy

- never
- rarely
- often
- very often

Weight loss

- never
- rarely
- often
- very often

Non-invasive ventilation

- never
- rarely
- often
- very often

**Section D**

8) *Are you satisfied how you managed SDB patients?*

- yes
- no

9) *In your clinical practice do you perform the night pulse oximetry tests on a child*

- yes
- no

10) *In your clinical practice do you perform the poligraphy with monitoring complete cardiorespiratory tests on a child*

- yes
- no

11) *In your clinical practice do you perform the complete polysomnography with EEG tests on a child*

- yes
- no
